# Surveillance for West Nile, Dengue, and Chikungunya Virus Infections, Veneto Region, Italy, 2010

**DOI:** 10.3201/eid1804.110753

**Published:** 2012-04

**Authors:** Federico Gobbi, Luisa Barzon, Gioia Capelli, Andrea Angheben, Monia Pacenti, Giuseppina Napoletano, Cinzia Piovesan, Fabrizio Montarsi, Simone Martini, Roberto Rigoli, Anna M. Cattelan, Roberto Rinaldi, Mario Conforto, Francesca Russo, Giorgio Palù, Zeno Bisoffi, the Veneto Summer Fever Study Group

**Affiliations:** Ospedale Sacro Cuore-Don Calabria, Negrar, Verona, Italy (F. Gobbi, A. Angheben, Z. Bisoffi);; Università di Padova, Padua, Italy (L. Barzon, M. Pacenti, G. Palù);; Istituto Zooprofilattico Sperimentale delle Venezie, Legnaro, Padua (G. Capelli, F. Montarsi);; Unità Locale Sanitaria 20–Regione Veneto, Verona (G. Napoletano);; Unità Locale Sanitaria 9–Treviso, Treviso, Italy (C. Piovesan); Entostudio, Brugine, Padua (S. Martini);; Ospedale Cà Foncello, Treviso (R. Rigoli);; Ospedale di Rovigo, Rovigo, Italy (A. M. Cattelan);; Ospedale di Padova, Padua (R. Rinaldi);; Ospedale San Bortolo, Vicenza, Italy (M. Conforto);; Regione Veneto–Servizio Promozione e Sviluppo Igiene e Sanità Pubblica, Venice, Italy (F. Russo)

**Keywords:** West Nile virus, dengue virus, chikungunya virus, summer fever, surveillance, Italy, viruses, vector-borne infections, arboviruses

## Abstract

In 2010, in Veneto Region, Italy, surveillance of summer fevers was conducted to promptly identify autochthonous cases of West Nile fever and increase detection of imported dengue and chikungunya in travelers. Surveillance highlighted the need to modify case definitions, train physicians, and when a case is identified, implement vector control measures

In 2010, a special surveillance for West Nile virus (WNV), dengue virus (DENV), and chikungunya virus (CHIKV) was initiated in the Veneto Region of northeastern Italy. The surveillance had 2 main objectives. First, we aimed to increase the detection rate of imported chikungunya and dengue in travelers from areas to which these diseases are endemic, including in new immigrants and settled immigrants visiting relatives and friends, and to promptly identify potential autochthonous cases. Second, we aimed to detect autochthonous cases of West Nile fever (WNF) and West Nile neuroinvasive disease (WNND), which were already included in regular surveillance, to acquire a more reliable picture of disease transmission in the region.

## The Study

In accordance with the study protocol, possible cases detected by general physicians and emergency department physicians had to be referred within 24 hours to the closest Unit of Infectious or Tropical Diseases. Serum samples from persons with possible cases were sent to the regional reference laboratory (Padua, Italy) for confirmation. If neuroinvasive disease was present, the specific protocol for WNND was followed (*1*).

**Figure 1:**
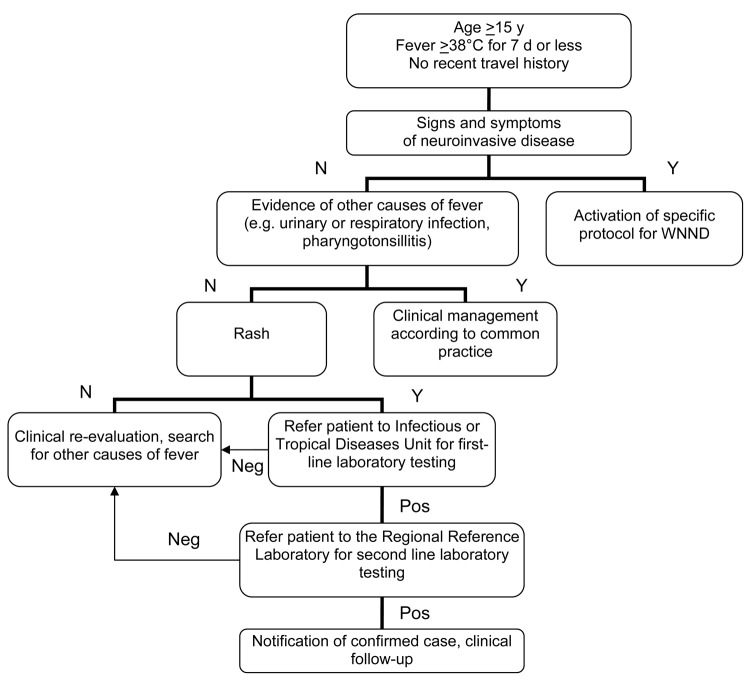
Algorithm for detection of possible cases of West Nile fever, Veneto Region, Italy, 2010. N, no; Y, yes; WNND, West Nile neuroinvasive disease; neg, negative; pos, positive.

We defined a possible case of DENV or CHIKV infection as fever >38°C during the past 7 days in a traveler who had returned within the previous 15 days from countries to which these viruses are endemic, absence of leucocytosis (leukocyte count <10,000 μL), and absence of other obvious causes of fever. After malaria was ruled out, cases were further classified as probable if rapid tests yielded positive results for dengue and chikungunya viruses. Rapid tests included detection of anti-CHIKV IgM by using the OnSite Chikungunya IgM Combo Rapid Test (CTK Biotech, Inc., San Diego, CA, USA) and of DENV nonstructural protein (NS) 1 antigen by using the Dengue NS1 Ag STRIP (Bio-Rad Laboratories, Hercules, CA, USA) on serum samples. Samples from persons with possible cases were sent to the regional reference laboratory for second-line laboratory testing and confirmation. Second-line laboratory testing consisted of detection of DENV and CHIKV nucleic acids in plasma specimens by using real-time PCR and endpoint PCR, respectively, and detection of serum IgM and IgG by using an anti-CHIKV indirect immunofluorescence assay (Euroimmun AG, Lübeck, Germany), DENV IgG DxSelect (Focus Diagnostics, Cypress, CA, USA), and DENV IgM Capture DxSelect (Focus Diagnostics). Samples with DENV -positive results by ELISA were further tested by plaque-reduction neutralization test to confirm specificity of antibody response. Confirmed cases were defined as the presence of viral nucleic acid in blood specimens or by seroconversion or detection of increasing serum levels of specific IgM and IgG. Possible autochthonous cases of WNF were defined as fever >38°C for <7 days, age >15 years, no recent travel history, rash, and absence of other obvious causes of fever (Figure 1).

In instances of high clinical suspicion for DENV and CHIKV in patients with autochthonous fever, laboratory tests for these 2 diseases also were performed. Moreover, all travelers tested for DENV and CHIKV also were tested for WNV.

Of 79 possible cases, we detected 14 cases of DENV infection and 1 case of CHIKV infection among travelers with fever (Table; Table A1). No cases were severe.

Four (11%) of 38 possible cases of autochthonous WNF were confirmed. All were positive for WNV IgM and/or IgG and confirmed by plaque-reduction neutralization test, but none were WNV RNA positive. Clinical descriptions of WNF and WNND cases are reported elsewhere (*1*).

## Conclusions

DENV, CHIKV, and WNV infections are arboviral diseases that find potentially suitable vectors in Italy, particularly in Veneto. No autochtonous case of fever caused by DENV has been documented in Italy, but the possible role of the *Aedes albopictus* mosquito as a vector has been demonstrated by recent cases in France (*2*) and Croatia (*3*).

CHIKV caused the well-known outbreak in Emilia Romagna Region (northern Italy) in 2007, which was detected, by coincidence a few days after the imported cases in Italy had been reported (*4*); the published report concluded that “the possibility of introducing CHIKV into Italy cannot be ruled out on the basis of current evidence.” The index case had occurred ≈2 months before the first case was diagnosed (*5*). The recent occurrence of 2 locally transmitted cases of chikungunya in France, despite a low number of imported cases (*6*), shows that the risk remains high.

Since summer 2008, WNV has caused WNND in humans, first in Emilia Romagna Region (*7*), then in Veneto Region (*8*). In contrast, the more common presentation, WNF, has been detected in only 1 patient; the casewas identified retrospectively (*9**,**10*), despite the expected WNF:WNND ratio of 20:1 (*11*).

Because we were concerned about being overwhelmed by an unmanageable number of case reports of unspecific fevers, we chose a selective case definition, particularly for WNF, with the obligatory presence of a rash, and thereby lowered the sensitivity of the surveillance. However, the proportion of virus-positive patients was strikingly high: ≈20% of persons tested who had imported fever were positive for DENV or CHIKV, as were 10% of persons with locally acquired fevers for WNV. Compared with the 2 previous years, the special surveillance enabled detection of substantially more cases, showing that you only find what you are looking for (Table). WNV circulation has now been documented in many areas of Italy, from north to south, through retrospective screening of solid organ donors (*12*) and through entomologic (*13*) and animal surveillance (*14*); nevertheless, in 2010, no human clinical cases were detected outside Veneto.

**Figure 2:**
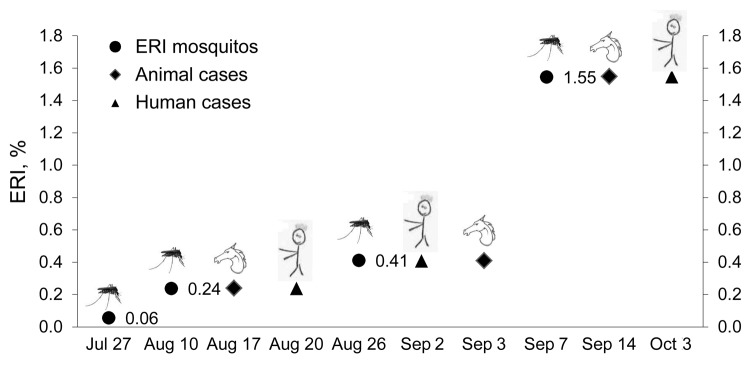
Expected rates of infection (ERI) in mosquitoes in the West Nile virus–positive site and hypothetical time from exposure to infected mosquitoes to clinical cases in animals and humans (calculated 1 week before symptom onset) recorded in the same province, Venice Province, Italy, 2010.

The success of this pilot phase prompted regional authorities to propose a 3-year plan, which the Ministry of Health has approved and funded, as part of the integrated surveillance of arboviral diseases, along with animal and entomologic surveillance. Relying only on the latter 2 would not be sensible. However, mosquito surveillance was able to predict cases in animals and humans (Figure 2). Expected rates of WNV infection in mosquitoes at the only site with repeated positivity in animals, humans, and vectors (Venice Province) are shown together with the time of exposure of animals and humans in the same province. Time of exposure was estimated as 1 week before onset of symptoms (incubation range 2–14 days) (*15*). When the expected rate of mosquito infection was low (i.e., 0.06%), no clinical cases were recorded; when the expected rate of infection was higher (>0.24%), clinical cases were observed in animals and humans.

Concerning the new plan for human surveillance of summer fevers, the case definition, particularly for WNF, has been modified by removing the compulsory presence of rash, to enhance sensitivity. Training and sensitization of general practitioners and emergency department physicians play a fundamental role. On the basis of a predefined threshold of vector intensity in an area where a new case has been identified, immediate vector control measures will be started when necessary.

## Appendix

**Table A1:** Description of 14 dengue cases and 1 chikungunya case, Veneto Region, Italy, 2010*

Infection/patient sex/age, y	Country of exposure	Time from symptom onset to diagnosis, d	Symptoms and signs							Laboratory results				Serotype
			Fever	Head ache	Joint pain	Muscle pain	Rash	Vomiting	Lymphadenopathy	Rapid test	IgG	IgM	PCR	
Dengue														
F/24	Bali	1	+	–	+	+	–	–	–	+	–	+	+	3
M/34	India	4	+	+	+	+	+	+	–	+	+	+	+	
M/38	French Guyana	NA	+	+	+	+	+	–	–	ND	+	+	+	
M/42	Cote d’Ivoire	3	+	–	+	–	–	–	–	+	–	–	+	3
M/43	Vietnam	5	+	+	+	–	+	–	–	+	–	+	+	2
F/43	Cambodia	3	+	+	+	+	–	–	+	+	–	–	+	2
F/31	Caribbean	10	+	+	–	–	+	–	–	+	+	+	+	1
M/35	Lao People’s Democratic Republic	4	+	+	+	+	+	–	–	+	–	+	+	2
M/40	Bangladesh	13	+	–	–	–	–	+	–	–	+	+	–	
F/51	India	7	+	+	–	–	–	–	–	+	+	+	–	
M/44	Thailand	7	+	+	+	–	–	–	–	+	+	+	–	
F/17	Martinica	6	+	+	+	+	–	–	–	–	+	+	–	
M/36	Thailand	20	+	+	–	–	+	–	–	–	–	+	–	
M/25	India	NA	+	–	–	–	–	–		+	–	+	+	
Chikungunya, F/58	Bali	NA	+	–	+	+	+	–	–	ND	+	+	–	

*+, positive; –, negative; NA, not applicable; ND, not done.

**Table:** Reported cases of West Nile, dengue, and chikungunya virus infections, Veneto Region, Italy, June 15–October 31, 2008–2010*

Year	Autochthonous WNF cases	Autochthonous WNND cases	Imported dengue cases	Imported chikungunya cases
2008	1 (retrospective)	5 (4 retrospective)	2	1
2009	0	6 (1 fatal)	4	0
2010†	4	3	14	1

*WNF, West Nile fever; WNND, West Nile neuroinvasive disease. †Surveillance started during the last week of July.
